# Ultra-high resolution 3D MRI for chondrocalcinosis detection in the knee—a prospective diagnostic accuracy study comparing 7-tesla and 3-tesla MRI with CT

**DOI:** 10.1007/s00330-021-08062-x

**Published:** 2021-05-28

**Authors:** Christoph Germann, Julien Galley, Anna L. Falkowski, Sandro F. Fucentese, Christian W. A. Pfirrmann, Daniel Nanz, Reto Sutter

**Affiliations:** 1 Department of Radiology, Balgrist University Hospital, University of Zurich, Zurich, Switzerland; 2 Department of Orthopedic Surgery, Balgrist University Hospital, University of Zurich, Zurich, Switzerland; 3 Swiss Center for Musculoskeletal Imaging (SCMI), Balgrist Campus AG, Zurich, Switzerland

**Keywords:** Magnetic resonance imaging, Multidetector computed tomography, Chondrocalcinosis, Knee joint

## Abstract

**Objectives:**

To test the diagnostic accuracy of a 3D dual-echo steady-state (DESS) sequence at 7-T MRI regarding the detection of chondral calcific deposits of the knee in comparison to 3-T MRI, using CT as cross-sectional imaging reference standard.

**Methods:**

CT and 7-T MRI (DESS) of knee joints in 42 patients with radiographically known chondrocalcinosis (13 of 42 bilateral) were prospectively acquired for all included patients (n = 55 knee joints). Additionally, 3-T MRI (DESS) was performed for 20 of these 55 knee joints. Two fellowship-trained musculoskeletal radiologists scored eight cartilage regions of each knee joint separately regarding presence of cartilage calcification, diagnostic confidence level, and sharpness of calcific deposits. In an explorative subanalysis, micro-CT of the menisci was evaluated after knee arthroplasty in one patient. Diagnostic performance metrics and nonparametric tests were used to compare between modalities. *p* values < 0.05 were considered to represent statistical significance.

**Results:**

Sensitivity for chondrocalcinosis detection was significantly higher for 7-T MRI (100%) compared to 3-T MRI (reader 1: 95.9%, *p* = 0.03; reader 2: 93.2%, *p* = 0.002). The diagnostic confidence was significantly higher for both readers at 7 T compared to both 3-T MRI (*p* < 0.001) and to CT (*p* = 0.03). The delineation of chondral calcifications was significantly sharper for 7-T compared to both 3-T MRI and CT (*p* < 0.001, both readers). Micro-CT in one patient suggested that 7-T MRI may potentially outperform standard CT in diagnosing chondral calcifications.

**Conclusion:**

3D-DESS imaging at 7-T MRI offers a significantly higher sensitivity in detection of chondral calcific deposits compared to 3-T MRI.

**Key Points:**

*• 3D dual-echo steady-state (DESS) MRI at 7 T has a higher sensitivity in detection of chondral calcific deposits compared to 3-T MRI (p ≤ 0.03).*

*• 3D DESS MRI at 7 T yields no false-negative cases regarding presence of chondral calcific deposits.*

*• 3D DESS MRI at 7 T offers better delineation and higher diagnostic confidence in detection of chondral calcific deposits compared to 3-T MRI (p < 0.001).*

**Supplementary Information:**

The online version contains supplementary material available at 10.1007/s00330-021-08062-x.

## Introduction

“Chondrocalcinosis” refers to the radiographic correlate of calcium-containing crystals (CaCs) which are deposited in the hyaline and/or fibrocartilage [1, 2]. Two main types of calcium-containing crystals exist: basic calcium phosphate (BCP) and calcium pyrophosphate (CPP). CPP can cause a crystal-induced arthropathy referred to as calcium pyrophosphate deposition disease (CPPD), a frequent cause of inflammation and pain known as pseudo-gout, commonly affecting the knee joint, followed by the wrist [3, 4]. Its prevalence is estimated to be 4–7% and represents a disease of aging, being rare in patients younger than 60 years of age [5, 6]. However, the epidemiological data may include a significant number of False-Positives, since it is largely based on radiographically detected chondrocalcinosis, which cannot differentiate non-CPP minerals such as BCPs from CPP-caused chondrocalcinosis [7]. On the other hand, absence of chondrocalcinosis does not rule out CPPD, as the acute arthropathy can precede radiographically detectable cartilage calcification [8–10].

It has been shown that chondral CaCs are associated with joint degeneration; however, it remains unclear whether chondrocalcinosis predisposes to or results from osteoarthritis [2, 11–15]. The imaging modalities used for detection of calcifications in the articular cartilage and menisci are usually X-ray-based, i.e., conventional radiographs and CT. Conventional radiographs have a higher spatial resolution; however, calcifications may either not be dense enough to be visualized, or it may be difficult to identify chondral CaC in severely degenerated joints [16, 17]. Furthermore, slightly rotated conventional radiographs may obscure the hyaline cartilage layer in certain areas of the knee joint, in particular the patellofemoral compartment and the posterior circumference of the femoral condyles. In contrast, CT facilitates chondrocalcinosis detection with a high contrast between calcifications and cartilaginous structures, and—due to the tomographic nature—allows a calcification assessment in all compartments and also in severely degenerated joints. Using MRI, gradient-echo sequences allow the visualization of chondrocalcinosis due to the sparsity of MR-visible protons in the crystals and due to crystal-induced local susceptibility effects, which result in well circumscribed signal losses [2, 18, 19]. Due to higher signal and more pronounced local susceptibility, ultra-high-field MRI potentially offers better CaC visualization compared to lower field strengths and potentially may even outperform radiographic modalities in calcification detection.

Therefore, the purpose of this study was to investigate the diagnostic accuracy and diagnostic confidence of a 3D-DESS sequence at 7-T MRI to detect chondral calcific deposits in all cartilaginous regions of the knee joint in comparison to 3-T MRI, using CT as current three-dimensional imaging standard.

## Materials and methods

This prospective single-center cohort study was approved by the local ethics committee (Cantonal Ethics Committee, Zurich). Written informed consent was given by all included subjects.

### Clarification of nomenclature

In this manuscript, we use the term “cartilage” as an umbrella term comprising both hyaline articular cartilage and fibrocartilage (menisci).

### Study population

**Fig. 1:**
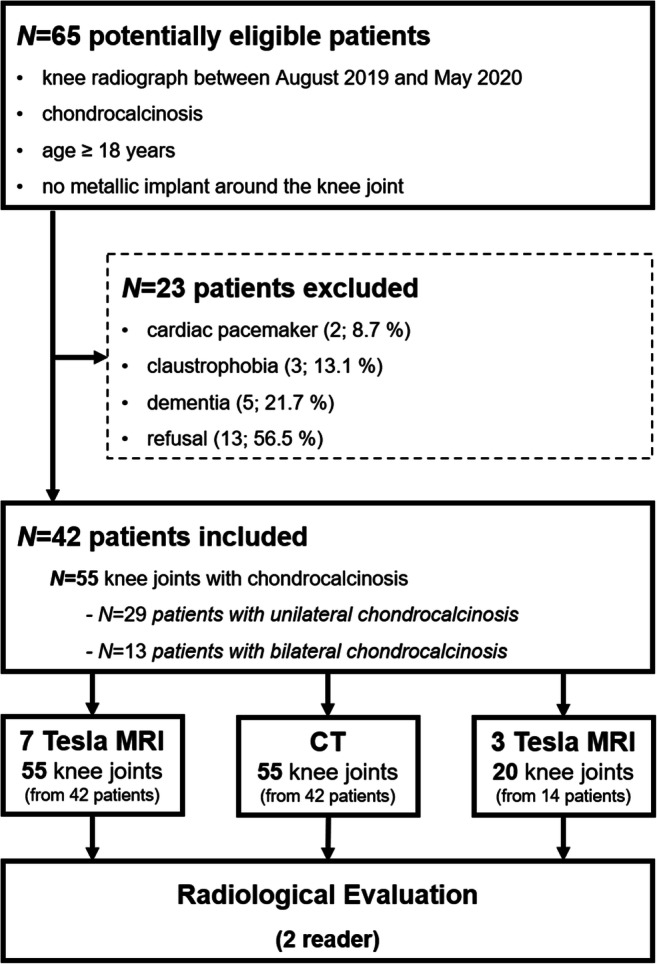
Flowchart of the study design and subjects

All knee radiographs acquired in our institution between August 2019 and May 2020 were screened for presence of chondrocalcinosis by a board-certified fellowship-trained musculoskeletal radiologist (C.G. with 7 years of experience). If CaCs were detected, the patient was asked to participate in the study. Inclusion criteria comprised (1) chondrocalcinosis present on radiographs of the knee joint, (2) age ≥ 18 years, and (3) no metallic implant around the knee joint. Exclusion criteria were as follows: (1) general contraindications for MRI (e.g., cardiac pacemaker); (2) claustrophobia; and (3) dementia. The flowchart of the study design is presented in Fig. 1.

### Imaging reference standard: CT

CT was chosen as imaging reference standard for detection of calcification based on (1) the high contrast between calcifications and cartilage and (2) the three-dimensional tomographic nature, thereby offering a more appropriate reference standard (as opposed to two-dimensional radiographs) when evaluating MRI as index test. After inclusion, each patient underwent a CT of the respective knee joint on a 128-detector row CT scanner (SOMATOM Edge Plus, Siemens Healthcare). The image datasets were acquired with a slice thickness of 0.5 mm and a pitch of 0.8, followed by a reconstruction in the axial (2 mm), coronal (2 mm), and sagittal plane (2 mm) using a dedicated bone kernel (Br60). Advanced Modeled Iterative Reconstruction (ADMIRE) level 3 was used for image reconstructions.

### MRI

All included subjects underwent an MRI examination of the respective knee joint on a 7-T MRI system (MAGNETOM Terra, Siemens Healthcare) with a dedicated 28-channel transmit/receive knee coil. Additionally, each patient was asked to further undergo a 3-T MRI examination (MAGNETOM Prisma, Siemens Healthcare) with a 15-channel transmit/receive knee coil. CT and MRI examinations were performed—whenever possible—on the same day. On both MRI scanners, a 3D DESS sequence was acquired. The detailed imaging parameters are listed in Table 1. The sequence parameters were optimized by scans with phantoms and asymptomatic volunteers. During this testing phase, it was attempted to reach the respective system-specific maximum isotropic spatial resolution, given a sufficient SNR within a clinically acceptable scan duration, comparable between 3-T and 7-T MRI. Limitations, such as the maximum flip angle due to the specific absorption rate (SAR) at 7 T, prevented to construct two fully identical pulse sequences at both field strengths. However, in order to assure the best attainable comparability between 7 and 3 T, it was attempted to keep the following parameters identical or as similar as possible: the acquisition time, the echo (TE) and repetition (TR) times, and the field of view. At 7 T, the maximum excitation flip angle compatible with SAR limitations was chosen.

**Table 1:** 3D dual-echo steady-state (DESS) imaging parameters for 3 T and 7 T. *TE* echo time, *TR* repetition time

Parameters	3 T	7 T
TR/TE (ms)	10.2/3.9	8/2.4
Excitation flip angle (degrees)	25	20
Field of view (mm) (readout/phase/slab direction)	160/160/111	160/160/128
Encoded voxel dimensions (mm)	0.63 × 0.63 × 0.63	0.40 × 0.40 × 0.50
Readout bandwidth (Hz/pixel)	455	695
Acquisition time (min:s)	07:38	07:42
Parallel-imaging acceleration factor	2	2
Acquisition orientation	Sagittal	Sagittal

### Ex vivo micro-CT

Based on the presumption that standard in vivo CT may miss small amounts/concentrations of chondral calcifications (false-negatives), an explorative ex vivo micro-CT of the menisci was performed for one patient after tissue-harvesting during a knee arthroplasty using a Skyscan 1176CT scanner (Skyscan) to obtain a high-resolution depiction of calcifications as ultimate reference standard. The scan parameters are as follows: 0.5 mm aluminum filter, voxel size of 35 μm, x-ray source voltage of 50 kV, current of 500 μA, exposure time of 80 ms, and rotation angle of 16°. Reconstruction was performed with DataViewer Software 1.4.4 (Skyscan).

### Image analysis

All CT and MR images were evaluated independently by two board-certified musculoskeletal radiologists (C.G. with 7 years and A.F. with 9 years of experience, respectively), blinded to all clinical data: image analysis was performed in a randomized fashion on anonymized data sets using a state-of-the-art picture archiving and communication system (Merlin, Phönix-PACS). The CT datasets were evaluated three months prior to the MR images.

In each knee joint, the following eight regions were evaluated separately: (a) medial meniscus, (b) lateral meniscus, (c) medial tibial plateau, (d) lateral tibial plateau, (e) medial femoral condyle, (f) lateral femoral condyle, (g) trochlea, and (h) patella. Based on every CT and MR image dataset, each region was assigned a score regarding the presence of calcifications in the cartilage (yes or no). In order to be classified as a chondral calcification, the image needed to show a clearly visible, unequivocal and distinct hyperdensity on CT, and marked hypointensity on MRI, located within the cartilage.

A 4-point Likert scale was used to express the diagnostic confidence regarding the presence of CaC, with grade 1, “not confident”; 2, “moderately confident”; 3, “quite confident”; and 4, “highly confident.” Additionally, the delineation of calcifications was graded on a 4-point Likert-scale: grade 1, “very sharp”; 2, “fairly sharp”; 3, “fairly fuzzy”; and 4, “very fuzzy.”

n = 440 regions with cartilage coverage were scored for CT and 7-T MRI (55 knee joints, eight different regions per knee joint) and n = 160 regions for 3-T MRI (20 knee joints, eight different regions per knee), respectively.

In case of disagreement between the two radiologists regarding calcifications being present or absent in the CT (two of 440 areas), a consensus reading between the two readers was performed together with a senior MSK radiologist (R.S. with 15 years of experience in musculoskeletal imaging) to ensure a common ground truth for CT to serve as the image reference standard for calcification detection.

Finally, each MRI exam was assessed for the presence of image artifacts (e.g., motion artifacts) by reader 1 (C.G.): grade 5 = “no artifacts,” 4 = “minimal artifacts,” 3 = “moderate artifacts, no impact expected on diagnostic value,” 2 = “pronounced artifacts, major impact on diagnostic value” and 1 = “severe artifacts, no diagnostic value.”

### Statistical analysis

Statistical analysis was performed using SPSS (v25, IBM Corp.) and MedCalc version 17.6 (MedCalc Software bvba). General descriptive statistics were applied. If not stated otherwise, categorical data are presented as proportions and percentages, ordinal data are given as median with range and continuous data as means with standard deviation. Interobserver reliability was assessed by calculating Cohen’s kappa for calcification detection (binary). Kappa values were interpreted according to Kundel and Polansky as either “slight” (0–0.20), “fair” (0.21–0.40), “moderate” (0.41–0.60), “substantial” (0.61–0.80), or “almost perfect” agreement (0.81–1.00) [20].

Sensitivities and specificities were compared using McNemar test. Receiver operating characteristic (ROC) curve analyses with calculation of the area under the curve (AUC) and 95% confidence interval (CI) were performed for the assessments of cartilage calcification for both readers on 3-T MRI und 7-T MRI. A statistically significant difference was accepted if the 95% CIs of the AUC did not overlap. McNemar test and Cochrane Q test were used to compare binary variables. Wilcoxon signed-rank test and Friedman test were applied to test for differences of ordinal variables. If applicable, post hoc analysis with Bonferroni method for multi-comparison correction was used. A *p* value < 0.05 was considered to represent statistical significance.

## Results

### Study group characteristics

Detailed demographics for all patients are given in supplemental Table 1. The study included a total of 42 patients with a mean age of 67 ± 10 years (range 46–91). Thirteen out of 42 patients had bilateral knee chondrocalcinosis with both knee joints having been examined for the study. Accordingly, our cohort comprised a total of 55 knee examinations for both CT and 7-T MRI; 20 of 55 examined knee joints were additionally examined by 3-T MRI, and the remaining 35 of 55 knee joints could not be examined at 3-T MRI due to participant refusal: the main reason for that was the high use of capacity of our 3-T MRI scanner which sometimes required the MRI scans at 7 T and 3 T to be performed on two different days and accordingly the participant to come to the hospital two times instead of only once. Of all included patients, 26 of 42 (62%) were male, and 16 of 42 (38%) female. Twenty-seven of 55 examined knees (49%) represented the left knee joint, and 28 of 55 (51%) the right knee joint, respectively. MR image quality assessment showed sufficient to mostly excellent image quality of all MRI datasets. 7 T/3 T: 7%/10% moderate artifacts, without impact on diagnostic value (grade 3); 15%/15% minimal artifacts (grade 4); 78%/75% no artifacts (grade 5).

### Frequency of findings

Detailed results are shown in Tables 2 and 3.

**Table 2:** Comparison of n = 20 knee examinations with all three modalities: CT, 3-T MRI, and 7-T MRI in regard to (a) cartilage calcification (no/yes), (b) diagnostic confidence level (1 = “not confident,” 2 = “moderately confident,” 3 = “quite confident,” 4 = “highly confident”), and (c) sharpness of calcifications (1 = “very fuzzy,” 2 = “fairly fuzzy,” 3 = “fairly sharp,” 4 = “very sharp”). Cochrane Q test (°) was applied for the item “cartilage calcification,” otherwise Friedman test (^**+**^) was used. *p* values < .05 are considered to represent statistical significance

Comparison of cartilage calcification between CT, 3-T MRI, and 7-T MRI						
Parameter		CT N = 20	3 T N = 20	7 T N = 20	*p* value °Cochrane Q test ^+^Friedman test	Multiple comparison analysis
Calcification (no/yes) n (%)	Reader 1	14/146 (9%/91%)	20/140 (13%/87%)*	13/147 (8%/92%)	0.008°	Different from CT and 7 T*
	Reader 2	14/146 (9%/91%)	24/136 (15%/85%)*	13/147 (8%/92%)	< 0.001°	Different from CT and 7 T*
Diagnostic confidence, median (range) 1 = not confident 4 = highly confident	Reader 1	4 (1–4)	4 (1–4)*	4 (1–4)	< 0.001^+^	Different from 7 T*
	Reader 2	4 (1–4)	4 (1–4)*	4 (1–4)	< 0.001^+^	Different from CT and 7 T*
Sharpness of calcification, median (range) 1 = very fuzzy 4 = very sharp	Reader 1	2 (1–4)	2 (1–4)	3 (1–4)*	< 0.001^+^	Different from CT and 3 T*
	Reader 2	2 (1–4)	2 (1–4)	4 (1–4)*	< 0.001^+^	Different from CT and 3 T*

*Statistically significant difference in the respective score, column “multiple comparison analysis” specifies between which two modalities the differences occur

**Table 3:** Comparison of n = 55 knee examinations at CT and 7-T MRI for both readers in regard to (a) presence of cartilage calcification (no/yes), (b) diagnostic confidence level (1 = “not confident,” 2 = “moderately confident,” 3 = “quite confident,” 4 = “highly confident”), and (c) sharpness of calcifications (1 = “very fuzzy,” 2 = “fairly fuzzy,” 3 = “fairly sharp,” 4 = “very sharp”). Mc Nemar test (°) was applied for the item “cartilage calcification”; otherwise, Wilcoxon signed-rank test (^**+**^) was used. *p* values < .05 are considered to represent statistical significance

Comparison of cartilage calcification between CT and 7-T MRI				
Parameter		CT N = 55	7 T N = 55	*p* value °Mc Nemar test ^+^Wilcoxon test
Calcification (no/yes) n (%)	Reader 1	86/354 (20%/80%)	54/386 (12%/88%)	< 0.001°
	Reader 2	86/354 (20%/80%)	48/392 (11%/89%)	< 0.001°
Diagnostic confidence, median (range) 1 = not confident 4 = highly confident	Reader 1	4 (1–4)	4 (1–4)	0.03^+^
	Reader 2	4 (1–4)	4 (1–4)	0.03^+^
Sharpness of calcification, median (range) 1 = very fuzzy 4 = very sharp	Reader 1	2 (1–4)	3 (1–4)	< 0.001^+^
	Reader 2	2 (1–4)	4 (1–4)	< 0.001^+^

#### Reference standard: CT

Based on the CT reference standard of all 55 knee joints (with a total of n = 440 different cartilage regions), 86 of 440 (19%) separately graded cartilage regions were classified as chondrocalcinosis-negative—representing “true-negatives”—whereas 354 of 440 (81%) regions were categorized as chondrocalcinosis-positive—hence representing “true-positives.” Accordingly, the CT evaluation of the 20 knee joints (with a total of n = 160 different cartilage regions) which have been subsequently scanned both at 3-T and 7-T MRI resulted in 14 of 160 (9%) chondrocalcinosis-negative regions as opposed to 146 of 160 (91%) chondrocalcinosis-positive regions. The diagnostic confidence for the calcification score was most frequently “high” for both readers (4: range 1–4). Additionally, both readers classified the calcifications mostly as “fairly sharp” (3: range 1–4).

#### 3-T MRI

Reader 1 classified 20/160 areas (13%) without cartilage calcification and accordingly 140 of 160 areas (87%) with calcification. Reader 2 found 24 of 160 areas (15%) with no cartilage calcification and 136 of 160 (85%) areas with calcification. The diagnostic confidence for the calcification score was most frequently “high” for both readers (4: range 1–4). Both readers classified the sharpness of calcifications mostly as “fairly fuzzy” (2: range 1–4).

#### 7-T MRI

Reader 1 scored 54 of 440 (12%) areas as having no cartilage calcification and accordingly 386 of 440 (88%) areas with calcification. Reader 2 graded 48 of 440 (11%) areas without cartilage calcification and 392 of 440 (89%) areas with calcification. The diagnostic confidence for the calcification score was most frequently “high” for both readers (4: range 1–4). Calcifications were graded most commonly as “fairly sharp” by reader 1 (3: range 1–4) and “very sharp” by reader 2 (4: range 1–4).

### Diagnostic performance regarding presence of chondral calcification

**Fig. 2:**
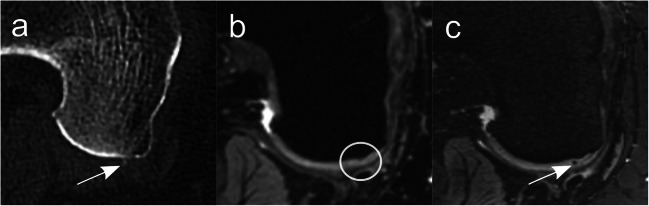
Spatially matched axial images (**a**: CT; **b**: DESS image at 3-T MRI; **c**: DESS image at 7-T MRI) of a 52-year-old man at the level of the posterior part of the medial femoral condyle of the right knee. Arrows highlight a clearly visible CaC deposit in the cartilage, seen as a point-shaped hyperdensity in CT (**a**) and as a point-shaped hypointensity in 7-T MRI (**c**) without a corresponding chondral signal change in the cartilage at 3-T MRI (**b**) (circle). *CaC* = calcium crystals, *DESS* = dual-echo steady state

Tables 4 and 5 show the diagnostic performance parameters of 3-T and 7-T MRI for diagnosing chondrocalcinosis. Sensitivity at 7-T MRI was 100% and was significantly higher for both readers compared to 95.9% (*p* = 0.03, reader 1) and 93.2% (*p* = 0.002, reader 2) at 3-T MRI. An example of a false-negative case for 3-T MRI in comparison to CT and 7-T MRI is shown in Fig. 2. The specificity and ROC AUC were numerically lower at 7-T MRI (specificity = 85.7%, both readers; ROC AUC = 0.93, both readers) compared to 3-T MRI (specificity = 100%, *p* = 0.5, both readers; ROC AUC = 0.98, *p* = 0.30 for reader 1 and ROC AUC = 0.97, *p *= 0.45 for reader 2), but without statistical significance.

**Table 4:** Contingency table of both readers for MRI diagnosis of the presence or absence of chondral knee calcification (reference standard: CT); upper part: contingency table for n = 160 separately analyzed chondral areas (3-T and 7-T MRI). Lower part: contingency table for n = 440 separately analyzed regions (7-T MRI)

		True-positive	False-positive	True-negative	False-negative
20 knee joints with a total of n = 160 separately analyzed regions					
3 T	Reader 1	140	0	14	6
	Reader 2	136	0	14	10
7 T	Reader 1	146	2	12	0
	Reader 2	146	2	12	0
55 knee joints with a total of n = 440 separately analyzed regions					
7 T	Reader 1	354	34	52	0
	Reader 2	354	38	48	0

**Table 5:** Sensitivity, specificity, and accuracy of both readers for MRI (either 3 T or 7 T) diagnosis of the presence or absence of chondral knee calcification (reference standard: CT)

		Sensitivity	Specificity	AUC
20 knee joints with a total of n = 160 separately analyzed regions				
3 T	Reader 1 (95% CI)	95.9% (90.9–98.3)	100% (73.2–100)	0.98 (0.96–1.0)
	Reader 2 (95% CI)	93.2% (87.4–96.5)	100% (73.2–100)	0.97 (0.94–0.99)
7 T	Reader 1 (95% CI)	100%* (96.8–100)	85.7% (56.2–97.5)	0.93 (0.82–1.0)
	Reader 2 (95% CI)	100%* (96.8–100)	85.7% (56.2–97.5)	0.93 (0.82–1.0)
55 knee joints with a total of n = 440 separately analyzed regions				
7 T	Reader 1 (95% CI)	100% (98.7–100)	60.5% (49.3–70.7)	0.82 (0.74–0.87)
	Reader 2 (95% CI)	100% (98.6–100)	55.8% (44.7–66.4)	0.78 (0.71–0.85)

*Significantly higher value for 7-T MRI in comparison to 3-T MRI for both readers (*p* = 0.03 for reader 1, *p* = 0.002 for reader 2, respectively)

*AUC* area under the receiver operating characteristic curve

### Comparison of diagnostic confidence and sharpness of chondral calcifications

#### CT vs 3-T MRI vs 7-T MRI (*n* = 20 knee joints)

**Fig. 3:**
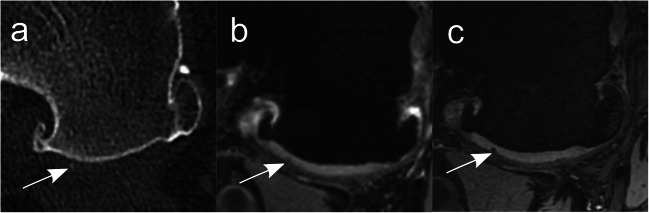
Differences in diagnostic confidence. Spatially matched axial images (**a**: CT; **b**: DESS image at 3 T MRI; **c**: DESS image at 7-T MRI) of a 51-year-old man at the level of the posterior part of the medial femoral condyle of the right knee. Arrows highlight a tiny, slightly hyperdense point-shaped chondral CaC deposit (**a**), with a clearly visible corresponding focal hypointensity at 7-T MRI (**c**) but with almost no equivalent signal change at 3-T MRI (**b**), accentuating the—in this case—considerably higher diagnostic confidence for detection of chondral CaC of 7-T MRI compared to 3-T MRI. Both readers scored the calcification as “quite confident” in CT (**a**), “not confident” in 3-T MRI (**b**) and “highly confident” in 7-T MRI (**c**). *CaC* = calcium crystals, *DESS* = dual-echo steady state

**Fig. 4:**
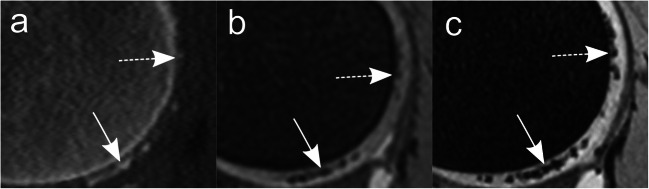
Difference in sharpness of chondral calcifications is illustrated on corresponding sagittal CT (**a**), 3-T MRI (**b**), and 7-T MRI (**c**) images of a 52-year-old woman depicting the posterior part of the right lateral femoral condyle. “Very fuzzy” CaCs are shown in the upper part of the femoral condyle as slight hyperdensities in CT (**a**) and slight hypointensities in 3-T MRI (**b**) compared to the “fairly sharp” circumscription of the corresponding clear-marked hypointensities in 7-T MRI (**c**) (dashed arrows). “Fairly sharp” confluent CaCs in the inferior part of the femoral condyle are shown in CT (**a**) and 3-T MRI (**b**), compared to a “very sharp” CaC distinction at 7-T MRI (**c**) (arrows). *CaC* = calcium crystals, *DESS* = dual-echo steady state

Detailed results are shown in Table 2. The diagnostic confidence for the presence of calcifications was significantly lower for 3-T MRI compared to 7-T MRI (reader 1: *p* = 0.007; reader 2: *p* < 0.001) (Fig. 3). Additionally, both readers had a lower diagnostic confidence level for 3-T MRI in comparison to CT images; however, a significant difference could be shown only for reader 2 (reader 1: *p* = 0.06; reader 2: *p* = 0.03). No difference in diagnostic confidence was identified for CT vs. 7-T MRI (reader 1: *p* = 1.0; reader 2: *p* = 0.51). The sharpness of calcifications was significantly better for 7 T compared to both 3-T MRI (both reader: *p* < 0.001) and CT (reader 1: *p* = .001; reader 2: *p* < 0.001) (Fig. 4). No significant difference was found for the sharpness of calcifications between CT and 3-T MRI (reader 1: 0 = 0.28; reader 2: *p* = 0.32).

#### CT vs 7-T MRI (n = 55 knee joints)

Detailed results are shown in Table 3. All cartilage calcifications detected at CT showed corresponding focal distinct hypointensities at 7-T MRI. The diagnostic confidence for the presence of calcifications was significantly higher at 7-T MRI compared to CT (both readers: *p* = 0.03). Furthermore, the sharpness of calcifications was significantly better for 7-T MRI compared to CT (both reader: *p* < 0.001).

### Explorative sub-analysis with ex vivo Micro-CT as ultimate reference standard (n = 1)

**Fig. 5:**
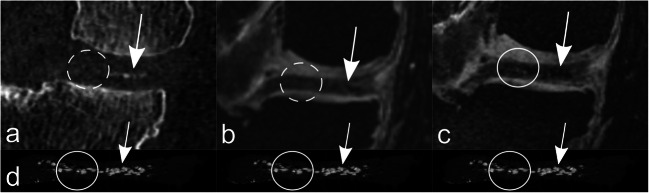
Spatially matched coronal CT (**a**), 3-T MRI (**b**), and 7-T MRI (**c**) images of the right medial femorotibial compartment of a 59-year-old man at the level of the posterior horn of the medial meniscus. The corresponding coronal micro-CT-image through the posterior horn of the medial meniscus is depicted (**d**)—for illustration purposes, the same micro-CT image is shown below each in vivo image. Arrows highlight visible CaC deposit in the medial part of the posterior horn of the medial meniscus in all modalities (**a**–**d**). In the lateral portion of the posterior horn of the medial meniscus are clearly discernible CaC deposits in the micro-CT image (**d**) and definite correlates at 7-T MRI (**c**) (circle); however, no correlation at 3-T MRI (**b**) and no certain correlation at standard in vivo CT (**a**) (dashed circles). *CaC* = calcium crystals, *DESS* = dual-echo steady state

Micro-CT images of a medial and lateral meniscus from one patient after resection during knee arthroplasty surgery were compared to the in vivo reference standard CT, as well as 3-T MRI and 7-T MRI. Few punctuate calcifications depicted in the micro-CT showed corresponding calcifications exclusively at 7-T MRI and not at reference standard CT or 3-T MRI (Fig. 5). Each chondral calcification at 7-T MRI had a corresponding visible calcification at Micro-CT.

### Interobserver reliability

The agreement between both readers for chondrocalcinosis detection was “almost perfect” for CT (κ = 0.99), 3-T MRI (κ = 0.90), and 7-T MRI (κ = 1.0).

## Discussion

This prospective study is the first to evaluate the potential role of ultra-high-field 7-T MRI compared to 3-T MRI and CT in detection of calcium crystal deposition in the cartilage. The dual-echo steady-state (DESS) sequence at 7-T MRI was found to be a valuable and precise tool to visualize chondrocalcinosis of the knee joint. With CT as current three-dimensional cross-sectional image standard for detection of soft tissue calcifications, we were able to demonstrate a significantly higher sensitivity for 7-T MRI (100%) compared to 3-T MRI. Furthermore, results of an additional explorative sub-analysis with micro-CT for one knee joint indicates that 7-T MRI may potentially offer superior visualization of calcium crystal deposits compared to standard in vivo CT.

Detection of chondrocalcinosis poses a diagnostic challenge. Traditionally, radiography is the standard imaging method used for the detection of intraarticular calcifications and features high spatial resolution. However, the projectional two-dimensional nature of radiographs leads to limited sensitivity, as chondral calcifications maybe obscured by the adjacent bone. Ultrasound yields a higher sensitivity compared to radiography [21] and similar to higher sensitivity compared to CT [22, 23]. However, the inability to assess all areas of cartilage within the joint and its operator dependency limits the general usefulness of ultrasound. CT has shown to be an accurate tool to assess soft tissue calcifications of the knee [24, 25] by combining the benefits of a cross-sectional imaging technique with multiplanar reconstruction, high resolution, and good soft tissue-mineralization contrast, thereby—in contrast to projection radiography—offering the ability to assess the whole cartilage of the knee joint. In regard to MRI, gradient echo-based sequences at high-field MRI (3 T and 4 T) have been shown to accurately depict cartilage calcifications and potentially even show a higher sensitivity compared to radiography [2, 19]. However, these studies only included four cases [19] or exclusively used radiography as imaging reference standard [2]. With the abovementioned advantages of CT, it seems more appropriate to analyze the diagnostic accuracy of MRI in calcification detection with CT serving as imaging reference standard. In contrast to the abovementioned studies [2, 19], Abreu et al examined ten cadaveric knee specimens (four of which with chondrocalcinosis) and found both histologic analysis and radiography to be more sensitive in calcification detection compared to MRI [26]. However, using low-field 1.5-Tesla MRI with only T1-weighted two-dimensional spin-echo sequences may explain the lower sensitivity compared to high-field MRI and/or 3D gradient echo sequences.

Due to its higher SNR, ultra-high-field 7-T MRI has the potential to increase the detection of soft tissue mineralization compared to lower field strengths. The findings of our study indicate a significantly higher sensitivity (100%) of ultra-high-field 7-T MRI compared to high-field 3-T MRI. Accordingly, if chondrocalcinosis was present, the respective chondral region was classified correctly by 7-T MRI (no false-negatives) as opposed to 3-T MRI (6 to 10 false-negatives). However, a numerically lower specificity was seen for 7-T MRI (85.7%) compared to 3-T MRI (100%), due to two false-positive classifications for 7-T MRI in 160 regions. In an explorative sub-analysis, high-resolution ex vivo micro-CT of the menisci for one patient was performed after receiving total knee arthroplasty, as micro-CT has been shown to be a valuable method to reliably depict and quantify small calcifications [26–29]: 7-T MRI was able to detect single focal meniscal CaC deposits that were only visible at high-resolution ex vivo micro-CT, but not at standard in vivo CT. This indicates that 7-T MRI may potentially have the ability to depict a smaller amount and/or lower concentration of cartilage mineralization compared to standard in vivo CT, and the actual false-positives in our diagnostic accuracy analysis for 7-T MRI may in fact (at least partly) represent true-positives, which are presumably not depicted by the current imaging standard CT. However, it is important to emphasize that this was an observation only in one patient and therefore not evidence-based.

Additionally, our findings demonstrate that 3-T MRI detected significantly fewer chondral calcifications compared to CT. This is in conflict with the findings of a retrospective study including 90 patients with knee chondrocalcinosis, demonstrating gradient-echo sequences at 3 T as a precise tool for cartilage calcification detection [2]. However, in that study, radiography served as reference standard, as opposed to our three-dimensional CT-based model. Furthermore, our results show a sharper appearance of CaC deposits and a higher diagnostic confidence for 7-T compared to both 3-T MRI and CT in regard to chondrocalcinosis detection.

Our study has limitations. First, mainly due to SAR and SNR limitations, the DESS sequence parameters were not completely identical between 3-T and 7-T MRI; however, within the inherent physical boundaries between the two field strengths, our sequences were designed to be as comparable as possible and make it appear unlikely that the minor parameter differences confound the results. Second, no “healthy” individuals as control group without chondrocalcinosis were included, as our study setting with separate analysis of eight different regions per knee joint was designed to provide the “negatives” (chondral regions without calcification) within the chondrocalcinosis cohort itself. The goal was to show differences regarding detection of chondral calcific deposits; therefore, the metrics in our study reflect the mere presence/absence of chondral calcific deposits in various regions of the knee joint, and not necessarily the difference in detecting general chondrocalcinosis within a whole knee joint. Third, only 20 knee joints (with a total of 160 separate chondral regions) could be examined at all three modalities (CT, 3-T MRI, and 7-T MRI), because more than half of the included patients refused the second MRI at 3 T. The main reason for this high refusal rate was the scanner availability: due to the high use of capacity (in particular the 3-T scanner), the MRI examinations had to be scheduled frequently on different days for 7 T and 3 T, which many participants did not concur with. Accordingly, more than half of the intended 3-T MRI could not be performed. However, by analyzing eight chondral regions per knee joint separately, it was possible to analyze a total of 160 regions.

In conclusion, a 3D DESS sequence at ultra-high-field 7-T MRI is a viable diagnostic test for detection of chondral calcific deposits, providing a high sensitivity with no false-negative cases, which was significantly better compared to 3-T MRI. Beyond that, DESS at 7-T MRI offers higher diagnostic confidence in chondrocalcinosis detection. Finally, future studies should address the diagnostic accuracy of 7-T MRI compared to high-resolution ex vivo micro-CT in detection of chondral calcific deposits, as our explorative sub-analysis indicates that some chondral calcifications (presumably small amounts and/or low concentration of calcium deposits in the cartilage) may be detected by 7-T MRI but not with standard in vivo CT.

## Supplementary information

ESM 1 (DOCX 16 kb)

## Abbreviations

ADMIRE: Advanced Modeled Iterative Reconstruction
AUC: Area under the curve
BCP: Basic calcium phosphate
CaC: Calcium crystals
CI: Confidence interval
CPP: Calcium pyrophosphate
CPPD: Calcium pyrophosphate deposition
DESS: Dual-echo steady state
PACS: Picture archiving and communication system
ROC: Receiver operating characteristic
SAR: Specific absorption rate
SNR: Signal-to-noise ratio
TE: Echo time
TR: Repetition time

## Declarations

### Guarantor

The scientific guarantor of this publication is Reto Sutter, MD.

### Statistics and biometry

No complex statistical methods were necessary for this paper.

### Methodology

• prospective

• diagnostic accuracy study

• performed at one institution
