# Supplementary material for: Ultra-high resolution 3D MRI for chondrocalcinosis detection in the knee—a prospective diagnostic accuracy study comparing 7-tesla and 3-tesla MRI with CT
Source: Eur Radiol. 2021 May 28;31(12):9436–45. doi: 10.1007/s00330-021-08062-x (PMC8589732; doi:10.1007/s00330-021-08062-x)
Supplement: Supplementary file 1 — (DOCX 16 kb) [file 330_2021_8062_MOESM1_ESM.docx]

|  | **n=42 patients**  **55 knee joints**  42 unilateral  13 bilateral | **n=14 patients**  **20 knee joints**  8 unilateral  6 bilateral |
| --- | --- | --- |
| **Age**, in years | 66.7 ± 10.3 | 62.7 ± 11.0 |
| **Sex**, n (%)  Male  Female | 26 (62 %)  16 (38 %) | 8 (57 %)  6 (43 %) |
| **Side**, n (%)  Right  Left | 28 (51 %)  27 (49 %) | 12 (60 %)  8 (40 %) |

**Supplemental Table 1.** Patient characteristics for patients who received 7 Tesla MRI and CT (n=42) and for patients who received 3 Tesla MRI and CT (n=14). Age is given as mean ± standard deviation. Sex and Side are given as absolute numbers (percentage in parenthesis).
